# A Numerical Model for Investigating the Effect of Viscoelasticity on the Partial Slip Solution

**DOI:** 10.3390/ma15155182

**Published:** 2022-07-26

**Authors:** Dongze Wang, Gregory de Boer, Ali Ghanbarzadeh

**Affiliations:** School of Mechanical Engineering, University of Leeds, Leeds LS29JT, UK; g.n.deboer@leeds.ac.uk (G.d.B.); a.ghanbarzadeh@leeds.ac.uk (A.G.)

**Keywords:** contact mechanics, coupled partial slip, viscoelasticity, friction

## Abstract

To investigate the effects of viscoelasticity on the stick-slip behaviour, a new model reproducing the partial slip of viscoelastic materials under fully coupled conditions is developed in this paper. The ratio of retardation time to relaxation time is employed to characterize the rheological property of a viscoelastic material. It is found that materials with higher ratios exhibit more fluid-like behaviours while those with lower ratios perform more like solid. As long as the contact input (load or displacement) is constant, the stick ratio (ratio of stick area to contacting area) is found to be insensitive to the viscoelasticity of materials. However, the separation pattern of the stick and slip regions varies with time when different contact phenomena (creep or stress relaxation) are encountered in the lateral and normal directions. The transition process from partial slip to gross sliding of viscoelastic materials, unlike the elastic response, tends to be abrupt when fully coupled conditions between shear tractions and pressure are introduced. When identical contact parameters are specified for different viscoelastic materials, the more fluid-like material always experiences a quicker transition from partial slip to gross sliding.

## 1. Introduction

Owing to the dimensional stability and capacity to sustain loads over long periods of time, viscoelastic materials have been extensively applied in many engineering fields, for example, as rubbers in automotive parts [1] and polymers in prosthetic joints [2]. When it comes to the design optimization and tribological analysis of these engineering components, the mechanical response of viscoelastic materials must be considered. However, a closed-form mathematical solution to viscoelastic contact problems is a significant challenge within the framework of classical contact mechanics as the structural complexity and time-dependent properties of these materials impede its development.

Great efforts have been made over the past few decades to provide solutions to various viscoelastic contact problems. Based on the assumption that the contact area increases monotonically without surface adhesion, a solution to the spherical indentation problem of linear viscoelastic materials was developed by Lee and Radok [3]. An essential concept known as the ‘elastic–viscoelastic correspondence principle’ was proposed in this study, in which the theory of Boltzmann hereditary integral is applied. This principle has now become the foundation for many recently developed analytical and numerical models of viscoelastic contact problems. The application of this tentative analytical method, including the limited contact geometry and monotony of the contact radius, was later extended somewhat by many researchers including Hunter [4], Graham [5], Yang [6], Ting [7,8], and Greenwood [9] using different approaches. A solution for the real contact area for a viscoelastic solid squeezed into a randomly rough surface was proposed by Persson et al. [10] using a contact-mechanics-based theory. However, these updated analytical solutions are still limited in the applications where they could be employed. Considering the existence of surface roughness, arbitrary loading history, and complicated rheological behaviour of viscoelastic materials in practical contact problems, it is difficult to obtain a valid time-varying solution based on existing analytical theories. A numerical model presents itself as a suitable alternative in this dilemma for which several simulation tools have been developed to assist in the analysis of viscoelastic components. A robust numerical model solving the point contact problem between a rigid indenter and a homogeneous viscoelastic half-space was presented by Chen et al. [11] using the semi-analytical method (SAM). Although the model was initially developed to simulate the normal and non-conformal contacts of polymer-based materials characterized by a wide spectrum of relaxation times and complicated surface topographies, it was extended to investigate more complicated contact problems. Yu et al. [12] included surface adhesion, Koumi et al. [13] considered heterogeneous viscoelastic materials and Spinu [14] analyzed the line contact configuration.

Apart from indentation problems, the sliding or rolling contact of viscoelastic materials has been one of the most investigated topics in the last few decades. Based on the correspondence principle, the rolling contact of a rigid cylinder on a viscoelastic half-space was analyzed by Hunter [15]. The solution to the rolling friction between a hard cylinder and a viscoelastic sphere was provided by Persson [16], and the sliding contact of a rigid wavy surface against a viscoelastic half-space was investigated by Menga et al. [17]. The geometrical limitations of the contacting bodies in these analytical solutions were overcome by numerical models developed later. Carbone and Putignano [18] proposed a novel method to analyze steady-state viscoelastic contact, in which a correction factor was introduced that takes into account the thickness of the contacting viscoelastic surface as well as the sliding speed. The model was employed to simulate the frictionless sliding contact of rough viscoelastic materials in different applications (half-space [19] and layer [20]). To further understand the complexity of viscoelastic sliding contact problems in practice, an ellipsoidal inhomogeneity was included by Koumi et al. [21] The rolling and sliding contacts of a layered viscoelastic half-space, in which the viscoelastic layer and substrate exhibit distinct properties, were simulated by Wallace et al. [22]. Meanwhile, the effects of interfacial imperfections between the layer and substrate on the transient and steady-state solutions were investigated by Zhang et al. [23].

Compared to the above-mentioned models of the frictionless sliding or rolling contacts of viscoelastic surfaces, less attention has been paid to the partial slip aspect of tangential contact problems. Under such a contact condition a global relative sliding does not occur. Instead, the contacting area is separated into stick zones, in which no relative motion happens, and slip zones, in which local relative movement exists. This type of contact phenomenon is commonly encountered in practice when two contacting surfaces are subjected to a tangential load that is not enough to induce gross sliding. An early viscoelastic partial slip solution was obtained by Goryacheva [24], where a viscoelastic cylinder rolling on a half-space of the same material was studied. Based on Coulomb’s friction law, the partial slip analysis on the rolling contact of viscoelastic multi-layered cylinders was provided by Kaller [25], whereas Goryacheva and Sadeghi [26] considered a different contact configuration (a cylinder sliding or rolling on a viscoelastic layer bonded to an elastic half-space) using the Goodman approximation [27].

The assumptions and approximations implemented in these tentative viscoelastic partial slip solutions become inapplicable when it comes to the contact of two dissimilar materials due to the significant interactions between normal pressures and shear tractions. Considering that a metal–polymer contact is one of the most common interfacial material combinations used in engineering practice, such as in medical devices [28], the coupling effects must be considered to obtain a valid solution. Numerical modelling of the coupled partial slip contact of viscoelastic materials was attempted by Spinu and Cerlinca [29], who considered the fretting of a steel sphere against a viscoelastic half-space under oscillating tangential loads. Based on the partial slip model of elastic materials by Gallego et al. [30], the stick–slip analysis in a frictional sliding contact of a rigid sphere on a viscoelastic layered half-space was presented in the work of Wallace et al. [22]. The partial slip periodic contact between a sinusoidal surface and a viscoelastic layer of finite thickness was analyzed by Bonari and Jacopo [31] using the finite element method (FEM), in which the coupling between the shear tractions in lateral directions was neglected. To date, the number of studies reporting the influence of viscoelasticity on the separation of stick and slip regions under fully coupled conditions is rather limited, and there is still a lack of understanding of the transition from partial slip to gross sliding in the field of viscoelastic contact. This is the problem we intend to address in the current paper.

By applying the elastic–viscoelastic correspondence principle, the novel conjugate gradient method (CGM)-based algorithm for the coupled normal and tangential contacts of dissimilar elastic materials, developed by the authors of the current manuscript [32], was extended here to obtain solutions to the stick–slip contact problems of viscoelastic surfaces. The numerical technique known as Discretized Convolution Fast Fourier Transform (DC-FFT) was implemented to improve the computational efficiency of the algorithm. The well-validated model was extended to investigate the effects of the time-dependent properties of viscoelastic materials on stick–slip solutions, where materials characterized by different rheological models were tested. A novel generalized view to understanding the responses of viscoelastic materials exhibiting different rheological properties is provided to explain why the shape of the contact tractions of a viscoelastic contact problem may vary significantly from the elastic solutions and why the unconventional transition process from partial slip to gross sliding occurs for viscoelastic materials. 

## 2. Theory and Algorithm Description

To explain how the viscoelastic model is developed, some of the basic principles about viscoelasticity are reviewed here for clarity. Viscoelastic materials exhibiting a linear relationship between stress and strain at any time point are known as linear viscoelastic materials. The viscoelastic surfaces being addressed in this work are assumed to behave linearly and the simulation of the nonlinear viscoelastic contact problems is beyond the scope of the current paper. Considering that viscoelastic materials are usually soft, they can hardly deform plastically. Therefore, plastic contact is not considered in the modelling work. In addition, the temperature during contact is assumed to be constant.

### 2.1. Theory of Linear Viscoelasticity

Within the framework of linear viscoelasticity, the responses of stress/strain to successive strain /stress stimuli are cumulative. The constitutive law of linear viscoelastic materials can be explained by two Heaviside step response functions to excitations known as the creep compliance function (Φ(*t*)) and relaxation modulus function (Ψ(*t*)). As their names suggest, the creep compliance function reveals the creep phenomenon and mathematically it describes the viscoelastic strain response to a unit change in stress. On the other hand, the relaxation modulus function characterizes the stress relaxation of viscoelastic materials and mathematically it represents the viscoelastic stress response to a unit change in strain. The Boltzmann hereditary integral is applied to characterize such a contact behaviour, where the response to sequential excitations can be the summation of the responses that would have been generated by each excitation applied alone. 

To consider the stress $\sigma_{1}\left(t\right)$ at time t under the acting of a certain strain $\Delta \varepsilon_{1}$ applied at the time $t_{1}^{\prime }$, it can be expressed as

(1) $$\sigma_{1}\left(t\right)=\Psi \left(t-t_{1}^{\prime }\right)\Delta \varepsilon_{1}$$

Likewise, the stress $\sigma_{2}\left(t\right)$ at time t under the performance of a strain $\Delta \varepsilon_{2}$ applied at $t_{2}^{\prime }$ is determined by

(2) $$\sigma_{2}\left(t\right)=\;\Psi \left(t-t_{2}^{\prime }\right)\Delta \varepsilon_{2}$$

Given a number of input strain increments that could be treated as a continuous distribution, the response of the strain to such an arbitrary sequence of strain becomes [33]

(3) $$\sigma \left(t\right)=\int_{0}^{t}\Psi \left(t-t^{\prime }\right)\;\frac{d\varepsilon \left(t^{\prime }\right)}{dt^{\prime }}dt^{\prime }$$

When the input quantity is the stress instead of the strain, the following relationship is obtained via an analogous derivation:

(4) $$\varepsilon \left(t\right)=\int_{0}^{t}\Phi \left(t-t^{\prime }\right)\frac{d\sigma \left(t^{\prime }\right)}{dt^{\prime }}dt^{\prime }$$

Unlike the case of an ideal elastic contact problem where the compliance and elastic modulus are mutually reciprocal, the relationship between the creep compliance and the relaxation modulus of viscoelastic materials is expressed as follows [34]:

(5) $$\int_{0}^{t}\Phi \left(t-t^{\prime }\right)\psi \left(t\right)dt^{\prime }=t$$

In the Laplace transform domain, there exists the following essential mathematical relationship [34]:

(6) $$\bar{\Phi }\left(s\right)\;\bar{\Psi }\left(s\right)\;=\;\frac{1}{s^{2}},$$

where s is the variable in the Laplace transform domain (s=a+jb). 

**Figure 1:**
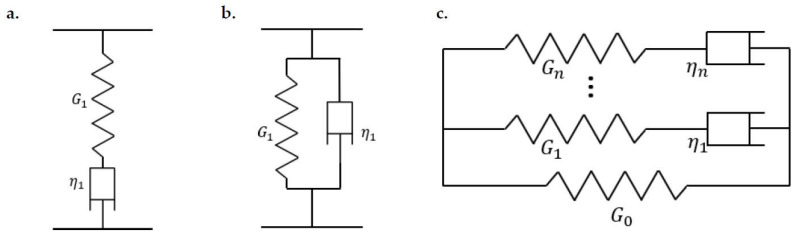
Rheological models describing the behaviour of linear viscoelastic materials: (**a**) Maxwell model, (**b**) Kelvin–Voigt model, and (**c**) generalized Weichert model: *G* is the spring stiffness and *η* is the dashpot viscosity.

Such time-dependent behaviours of linear viscoelastic materials can be expressed in terms of rheological models established with linear springs (perfectly elastic body) and dashpots (ideal Newtonian fluid). When a spring and a dashpot are arranged in a series as presented in Figure 1a, such a rheological model is known as the Maxwell model. The Kelvin–Voigt model is established if the two elements are arranged in parallel as illustrated in Figure 1b. It is acknowledged that a Maxwell model demonstrates the stress relaxation of linear viscoelastic materials appropriately, but it fails to account for their creep and recovery characteristics. On the contrary, the Kelvin–Voigt model performs oppositely. Furthermore, it exhibits no instantaneous elastic response. A detailed analysis of these two models can be found in the work of Popov [33]. Considering that these two-element models can only provide qualitative descriptions, a more sophisticated model, such as the generalized Weichert model, comprising more elements as shown in Figure 1c is usually used to characterize the linear viscoelastic materials with precise quantitative information. The number of elements in the model is determined by the naturally occurring spectrum of relaxation times, where the relaxation time is denoted by τ and determined by the properties of the elements as follows:

(7) $$\tau =\frac{\eta_{i}}{G_{i}},$$

where $\eta_{i}$ is the viscosity of the i-th dashpot and $G_{i}$ is the modulus of the *i*-th spring.

With the generalized Weichert model, the relaxation modulus function of any linear viscoelastic material can be expressed mathematically by fitting the experimental data collected from the conducted relaxation test to the following equation (Prony series [33]) and adjusting the parameters:

(8) $$\Psi \left(t\right)=G_{0}+\overset{n}{\underset{i=1}{\sum }}G_{i}\exp\left(-\frac{t}{\tau_{i}}\right)$$

It is noted that the spectrum of relaxation times of linear viscoelastic materials can be characterized by introducing as many exponential terms as needed to attain the expected accuracy of the curve fitting. Once the relaxation modulus function is available, the corresponding creep compliance function can be easily determined as well utilizing their mathematical relationship in the Laplace domain (Equation (6)). If the material is characterized by one relaxation time as in this case, such a three-element model is known as the Zener model or standard linear solid model. 

### 2.2. Problem Formulation

**Figure 2:**
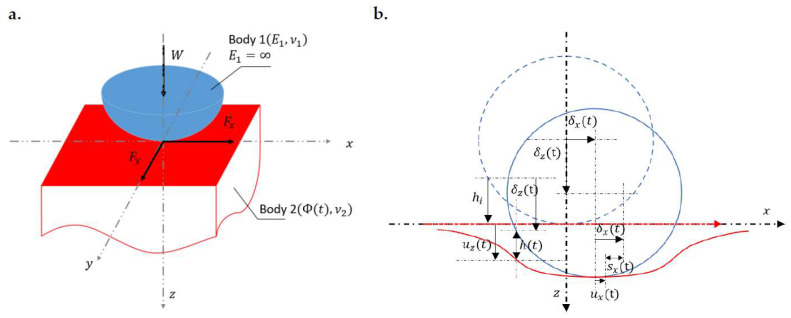
(**a**) Geometry of the contacting bodies: $W,\;\;F_{x},\;\;F_{y}$ are the input normal load and the tangential loads in *x* and *y* directions, respectively, $E_{1}$ ($E_{1}=\infty$) and $\nu_{1}$ are the elastic modulus and Poisson’s ratio of the rigid sphere, respectively, and Φ(t) and $\nu_{2}$ are the time-dependent creep compliance and Poisson’s ratio of the viscoelastic half-space, and (**b**) Transient displacement condition on the x−z plane: $h_{i}$ denotes the gap between surfaces before loading, $h\left(t\right),\;\;s_{x}\left(t\right),\;\;\delta \left(t\right),\;\;u\left(t\right)$ are the gap between deformed surfaces, the slip distance in the *x* direction, the rigid body displacement, and the surface deformation after a certain time t, respectively, with subscript denoting the direction of relevant vectors.

Although the time derivatives appearing in the governing equations (i.e., Equations (3) and (4)) add the third argument to the problem parameters and subsequently bring about complications when addressing the viscoelastic contact problems, the field equations still possess certain mathematical features. It makes the viscoelastic solution (i.e., the displacement response of linear viscoelastic materials to an arbitrary distribution of contact tractions) readily accessible instead of deriving it from scratch. A common practice is to adapt the existing well-developed elastic solutions to make them applicable to linear viscoelastic materials following the elastic–viscoelastic correspondence principle. Steps to take for the transition from elastic solutions to the viscoelastic counterparts when dealing with a typical half-space contact problem (a rigid sphere against a viscoelastic half-space as shown in Figure 2a) are detailed as follows:

1. replace the constant elastic contact compliance (1/2G) with the time-dependent creep compliance Φ(t);
2. subdivide the pressure history in the simulation time p(t) into infinitesimal intervals ($\frac{\partial p}{\partial t^{\prime }})$;
3. superpose the contributions of pressures in all subdivided time intervals by making use of the hereditary integral ($\int_{0}^{t}()dt^{\prime })$.

Based on the corresponding solution for an elastic material (the Boussinesq solution [35]), the normal displacement of the viscoelastic surface caused by the normal traction at the time t can be determined as follows:

(9) $$\begin{array}{c} u_{zz}^{e}\left(x_{1},x_{2}\right)=\int_{-\infty }^{\infty }\;\int_{-\infty }^{\infty }\;\frac{\left(1\;-\;\nu \right)p\left(x_{1}^{\prime },x_{2}^{\prime }\right)dx_{1}^{\prime }dx_{2}^{\prime }}{2\pi G\sqrt{{\left(x_{1}\;-\;x_{1}^{\prime }\right)}^{2}\;+\;{\left(x_{2}\;-\;x_{2}^{\prime }\right)}^{2}}};\\ u_{zz}^{e}\left(x_{1},x_{2}\right)=\int_{-\infty }^{\infty }\;\int_{-\infty }^{\infty }\;\;G^{e}\left(x_{1}-x_{1}^{\prime },x_{2}-x_{2}^{\prime }\right)p\left(x_{1}^{\prime },x_{2}^{\prime }\right)dx_{1}^{\prime }dx_{2}^{\prime }, \end{array}$$

(10) $$\begin{array}{r} u_{zz}^{v}\left(x_{1},x_{2},t\right)=\int_{0}^{t}\int_{\infty }^{\infty }\;\int_{\infty }^{\infty }\frac{\left(1\;-\;\nu \right)\Phi \left(t\;-\;t^{\prime }\right)}{\pi \sqrt{{\left(x_{1}\;-\;x_{1}^{\prime }\right)}^{2}\;+\;{\left(x_{2}\;-\;x_{2}^{\prime }\right)}^{2}}\;}\frac{\partial p\left(x_{1}^{\prime },x_{2}^{\prime },t^{\prime }\right)}{\partial t^{\prime }}dx_{1}^{\prime }dx_{2}^{\prime }dt^{\prime };\\ u_{zz}^{v}\left(x_{1},x_{2},t\right)=\int_{0}^{t}\int_{\infty }^{\infty }\;\int_{\infty }^{\infty }G_{zz}^{v}\left(x_{1}-x_{1}^{\prime },x_{2}-x_{2}^{\prime },t-t^{\prime }\right)\frac{\partial p\left(x_{1}^{\prime },x_{2}^{\prime },t^{\prime }\right)}{\partial t^{\prime }}dx_{1}^{\prime }dx_{2}^{\prime }dt^{\prime }, \end{array}$$

where Equation (9) is the Boussinesq integral for elastic surfaces and Equation (10) is the adapted viscoelastic solution based on the corresponding principle, in which the Green’s function $G_{zz}^{v}\left(x_{1},x_{2},t\right)$ characterizes the normal deformation at the point of coordinates $\left(x_{1},x_{2}\right)$ and at the time point t induced by a unit concentrated and normal force and it is determined as follows:

(11) $$G_{zz}^{v}\left(x_{1},x_{2},t\right)=\frac{\left(1-\nu \right)\Phi \left(t\right)}{\pi \sqrt{x_{1}^{2}+x_{2}^{2}}}$$

Through the manipulation of elastic solutions, all the continuous equations accounting for the effects of loading history on the surface displacements during the viscoelastic contact period can be obtained.

A proper discretization is a prerequisite for the development of a valid viscoelastic contact model. Apart from the spatial discretization where the area of interest between two contacting bodies is meshed into equally spaced rectangular elements with a size of 2a×2b and the numbers $N_{1}$ and $N_{2}$ in the x and y directions, respectively, supplementary temporal discretization is necessary to model the viscoelastic contact due to the aforementioned effects of the loading history on the viscoelastic deformation. The total simulation time T is thus discretized into $N_{t}$ time points. The time interval is Δt, which is uniform and assumed to be sufficiently short so that the elemental pressure is assumed to be constant. Based on the assumption, the partial derivative $\frac{\partial p\left(x_{1}^{\prime },x_{2}^{\prime },t\right)dt^{\prime }}{\partial t^{\prime }}$ in Equation (10) can be substituted by the finite pressure difference ‘p(i,j,k)−p(i,j,k−1)’, where p(i,j,k) is the discretized counterpart of p(x,y,t) with $i=1\ldots N_{1}$, $j=1\ldots N_{2}$, $t=k\Delta_{t}$, and $k=1\ldots N_{t}$.

As a result of the spatial and temporal discretizations, the continuous form of Equation (10) is modified into the following piecewise definition:

(12) $$u_{zz}^{v}\left(i,j,k\right)=\overset{N_{t}}{\underset{n=1}{\sum }}\overset{N_{1}}{\underset{l=1}{\sum }}\overset{N_{2}}{\underset{m=1}{\sum }}IC_{zz}^{v}\left(i-l,j-m,k-n\right)\left(p\left(l,m,n\right)-p\left(l,m,n-1\right)\right),\;$$

where $IC_{zz}^{v}\left(i-l,j-m,k-n\right)$ is known as the viscoelastic influence coefficient characterizing the normal displacement observed after k time steps in the node (*i, j*) of the spatial mesh under the effect of a uniform pressure of 1/(4ab) Pa acting on the node (*l, m*) in the *n*-th time step after the reference time, with n≤k. 

After determining the closed-form discretized solutions of all influence coefficient matrices relating the surface displacements and contact tractions in the *x*, *y,* and *z* directions, nodal displacements of a linear viscoelastic surface induced by an arbitrary history of nodal tractions can then be expressed as follows:

(13) $$\begin{array}{c} \begin{array}{c} u_{x}\left(i,j,k\right)\\ u_{y}\left(i,j,k\right)\\ u_{z}\left(i,j,k\right) \end{array}=\overset{N_{t}}{\underset{n=1}{\sum }}\overset{N_{1}}{\underset{l=1}{\sum }}\overset{N_{2}}{\underset{m=1}{\sum }}\;\left[\begin{array}{ccc} IC_{xx}^{v}\left(i-l,j-m,k-n\right) & IC_{xy}^{v}\left(i-l,j-m,k-n\right) & IC_{xz}^{v}\left(i-l,j-m,k-n\right)\\ IC_{yx}^{v}\left(i-l,j-m,k-n\right) & IC_{yy}^{v}\left(i-l,j-m,k-n\right) & IC_{yz}^{v}\left(i-l,j-m,k-n\right)\\ IC_{zx}^{v}\left(i-l,j-m,k-n\right) & IC_{zy}^{v}\left(i-l,j-m,k-n\right) & IC_{zz}^{v}\left(i-l,j-m,k-n\right) \end{array}\right]\\ \;\;\;\;\;\;\cdot \left[\begin{array}{c} q_{x}\left(l,m,n\right)-q_{x}\left(l,m,n-1\right)\\ q_{y}\left(l,m,n\right)-q_{y}\left(l,m,n-1\right)\\ p\left(l,m,n\right)-p\left(l,m,n-1\right) \end{array}\right] \end{array}$$

where DC-FFT is applied to accelerate the convolution operations. For the sake of simplicity, Coulomb’s friction law is implemented here to identify the stick and slip regions within the contacting area based on a constant and uniform coefficient of friction $\mu_{f}$. A quasi-static process is assumed to avoid the problem of dissipative friction and its irreversibility related to the load-path dependency when addressing the frictional contact problems. It is important to note that the Poisson’s ratio of a viscoelastic material employed in practice is usually time-dependent [36] but is assumed to be constant here for simplicity.

With the equations and assumptions described above, a sequence of discretized contact problems with complementary conditions can be constructed to search for the solution that accurately reproduces the history of the viscoelastic contact process. The boundary conditions in discretized viscoelastic contact problems are in an analogous form to the elastic ones summarized by Johnson [37], except that the time is now accounted for:

1. Load balance:

The sum of the traction distribution at the contact interface should be strictly equal to the input load in the corresponding direction at any specific time point during the discretized simulation period:

(14) $$\begin{array}{c} W\left(t\right)=\Delta \underset{\left(i,j\right)\in I_{c}\left(k\right)}{\sum }p\left(i,j,k\right),\;F_{x}\left(t\right)=\Delta \underset{\left(i,j\right)\in I_{c}\left(k\right)}{\sum }q_{x}\left(i,j,k\right),\;\\ F_{y}\left(t\right)=\Delta \underset{\left(i,j\right)\in I_{c}\left(k\right)}{\sum }q_{y}\left(i,j,k\right),\;k=1\ldots N_{t}, \end{array}$$

where Δ(2a×2b) is the area of each element in the set mesh and $I_{c}\left(k\right)$ is the contact domain at the k-th time point.

2. The deformation of viscoelastic surfaces must meet the following geometrical conditions at any specific time:

In the normal direction, the following condition of surface separation should be met:

(15) $$h\left(i,j,k\right)=h_{i}\left(i,j\right)+u_{z}\left(i,j,k\right)-\delta_{z}\left(k\right),\;k=1\ldots N_{t},\;\left(i,j\right)\in I_{p}$$

where $I_{p}$ denotes the whole simulation domain, $h_{i}$ is the gap between undeformed surface, and *h*, and $\delta_{z}$ are the gap between deformed surfaces and the normal rigid body displacement at the specified time point k, respectively, as illustrated in Figure 2b.

In the two tangential directions, the following displacement equation should be met:

(16) $$\begin{array}{c} s_{x}\left(i,j,k\right)=u_{x}\left(i,j,k\right)-\delta_{x}\left(k\right),\;\left(i,j\right)\in I_{c}\left(k\right);\\ s_{y}\left(i,j,k\right)=u_{y}\left(i,j,k\right)-\delta_{y}\left(k\right),\;\left(i,j\right)\in I_{c}\left(k\right), \end{array}$$

where $s_{x}$ and $s_{y}$ are, respectively, the slip distances in the *x* and *y* directions at a specific time point, whereas $\delta_{x}$ and $\delta_{y}$ denote the time-dependent rigid body displacements in the *x* and *y* directions, respectively, as shown in Figure 2b.

3. Complementary conditions should be satisfied at any specific time over the contacting surfaces:

In the normal direction, the product of the pressure and surface gap must always be zero over the whole computational domain $I_{p}$ expressed as:

(17) $$\begin{array}{c} p(i,j,k)\;>\;0\;\&\;h\left(i,j,k\right)=0,\;\left(i,j\right)\in I_{c}\left(k\right);\\ p\left(i,j,k\right)=0\;\&\;h\left(i,j\right)\;>\;0,\;\left(i,j\right)\in I_{p}-I_{c}\left(k\right), \end{array}$$

where $I_{p}-I_{c}\left(k\right)$ denotes the non-contacting domain at the time step k.

Equation (17) implies that the investigated surfaces are impenetrable and the surface adhesion is not considered in the given contact analysis.

In the lateral directions, the shear traction magnitude must be lower than the local friction and no slip should exist in the time-dependent stick zone according to Coulomb’s friction law. As to the slip region, the norm of the shear tractions must be equal to the local friction. In addition, the direction of the shear stress should be opposite to that of the slip distance. Such contact conditions are expressed as follows:

(18) $$\begin{array}{c} \sqrt{q_{x}{\left(i,j,k\right)}^{2}+q_{y}{\left(i,j,k\right)}^{2}}<\mu_{f}p\left(i,j,k\right),\;\left|s_{x}{\left(i,j,k\right)}^{2})+s_{y}{\left(i,j,k\right)}^{2}\right|=0,\;\;\left(i,j\right)\in I_{s}\left(k\right);\\ q_{x}\left(i,j,k\right)\cdot s_{x}\left(i,j,k\right)+q_{y}\left(i,j,k\right)\cdot s_{y}\left(i,j,k\right)<0,\;\left(i,j\right)\in I_{c}\left(k\right)-I_{s}\left(k\right);\\ \sqrt{q_{x}{\left(i,j,k\right)}^{2}+q_{y}{\left(i,j,k\right)}^{2}}=\mu p\left(i,j,k\right),\;\left|s_{x}{\left(i,j,k\right)}^{2})+s_{y}{\left(i,j,k\right)}^{2}\right|\neq 0,\;\left(i,j\right)\in I_{c}\left(k\right)-I_{s}\left(k\right). \end{array}$$

### 2.3. Algorithm Description

In order to search the time-dependent contact tractions and surface deflections meeting the conditions described above, CGM is adopted considering its guaranteed convergence for quadratic optimization problems with inequality constraints. For each time step, the corresponding surface gap g in the contacting area and slip distance s in the stick regions are the system residuals to be minimized in established numerical optimization problems. The normal and tangential contact problems are addressed in two separate solvers, in which the contribution of contact tractions in all directions is considered when determining the surface displacement.

**Figure 3:**
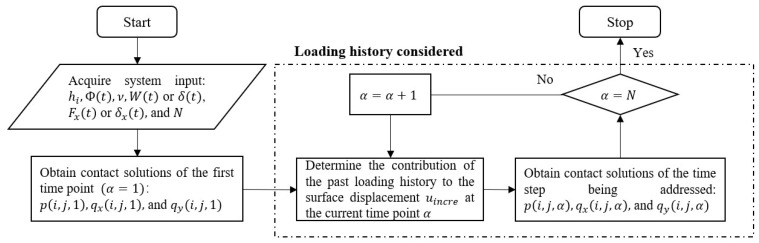
Flow chart of the algorithm for the viscoelastic partial slip contact problems.

When the loads or displacements are just applied at the first time point, there are no effects on the loading history. Hence, the viscoelastic contact problem being addressed temporarily becomes a time-independent coupled elastic one, for which the constant material property is the instantaneous modulus Ψ(0). The iterative process to obtain a coupled elastic solution is detailed in reference [32], after which the loading history is accounted for to obtain the contact solutions of the following time steps. The overall numerical approach to viscoelastic partial slip contact problems is shown in Figure 3. The effects of loading history are included by considering the contribution of the past loading history to the surface displacement. To consider the surface displacement in the normal direction derived from normal pressures $u_{zz}$ at time step α, the additional displacement component from the pressure history can be determined as follows:

(19) $$u_{incre}\left(i,j,\alpha \right)=\overset{\alpha -1}{\underset{n=1}{\sum }}\overset{N_{1}}{\underset{l=1}{\sum }}\overset{N_{2}}{\underset{m=1}{\sum }}\left(IC_{zz}^{v}\left(i-l,j-m,\alpha +1-n\right)\cdot p\left(l,m,n\right)-IC_{zz}^{v}\left(i-l,j-m,\alpha -n\right)\cdot p\left(l,m,n\right)\right)$$

According to Equation (19), the extra deformation for $u_{zz}$ at the second time point should be $‘IC_{zz}^{v}\left(:,:,2\right)*p\left(:,:,1\right)-IC_{zz}^{v}\left(:,:,1\right)*p\left(:,:,1\right)’$. The incremental deformations at the rest of the time points, as well as those arising from contact tractions in other directions, can be determined in an identical fashion, which is suggested to be executed outside the iteration process of the contact solvers to reduce the computational time.

After adjusting the geometrical conditions of deformation (Equations (15) and (16)) by converting the surface deformation u into the following form,

(20) $$u=u_{incre}+u_{t}$$

(21) $$u_{t}=IC\left(:,:,1\right)*t\left(:,:,\alpha \right)$$

the unknown terms in the new equations are reduced to the contact tractions at the time point being considered t(:,:,α) and the resulting displacement components $u_{t}$.

The viscoelastic stick–slip contact problem is thus transformed into a sequence of instantaneous elastic counterparts, for which an algorithm similar to that given in the work of Wang et al. [32] is employed to search for the solutions until the end of the simulation time is reached.

## 3. Model Validation

Due to the limited literature concerning the coupled partial slip of viscoelastic materials, the validation work for the developed model is separated into two separate sections, in which reference solutions are given. First, the coupled stick–slip aspect of the model has been validated by simulating the partial slip contact between a rigid sphere and an elastic half-space, of which a detailed description is given in the work of Wang et al. [32]. Second, the viscoelastic aspect of the model is validated by simulating the contact of a rigid sphere indenting a viscoelastic material and is detailed as follows.

To simulate the normal contact problem of a rigid sphere indenting an incompressible viscoelastic half-space, the time-dependent behaviour of the viscoelastic material is simplified and described by a Maxwell model in which the creep compliance and relaxation modulus are characterized as follows:

(22) $$\Phi \left(t\right)=\frac{1}{G}+\frac{t}{\eta }$$

(23) $$\Psi \left(t\right)=G\cdot \exp\left(-\frac{t}{\tau }\right)$$

The geometry and material properties of the contacting surfaces are given in Table 1.

A step load of 100 N is applied instantaneously at the beginning of the simulation window (t=0) and maintained throughout the whole contact period. The simulation results derived from the model are nondimensionalized by the Hertzian solutions of a normal sphere contact, where the instantaneous modulus Ψ(0) is the input material property. The time-dependent contact tractions and nodal coordinates are normalized by the peak normal pressure $p_{0}$ and contact radius $a_{0}$ (shown in Table 1), respectively. The contact solutions given hereinafter are all normalized in the same method if not mentioned otherwise. The potential contact area (512 μm×512 μm) is discretized by a mesh system with 256×256 uniformly distributed elements and the whole viscoelastic contact period (T=2τ) is subdivided into 101 time points. 

**Figure 4:**
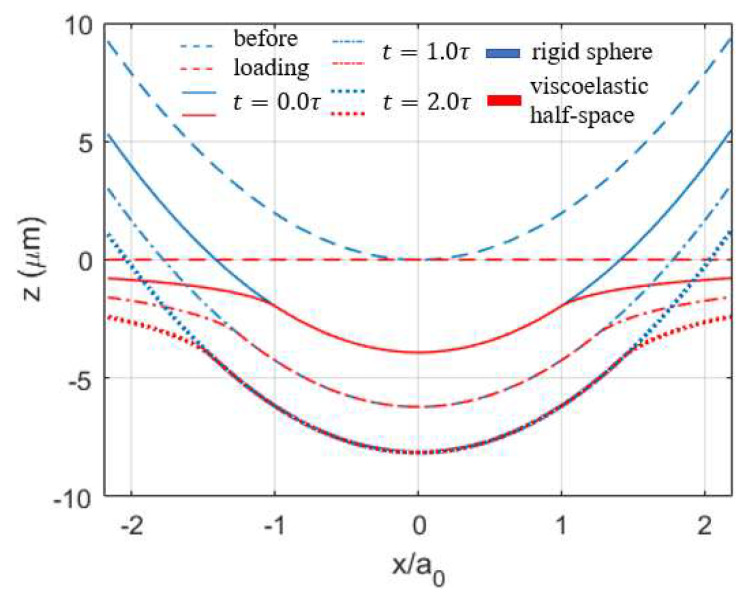
Creep of surface profile at different time points under the input normal load.

**Figure 5:**
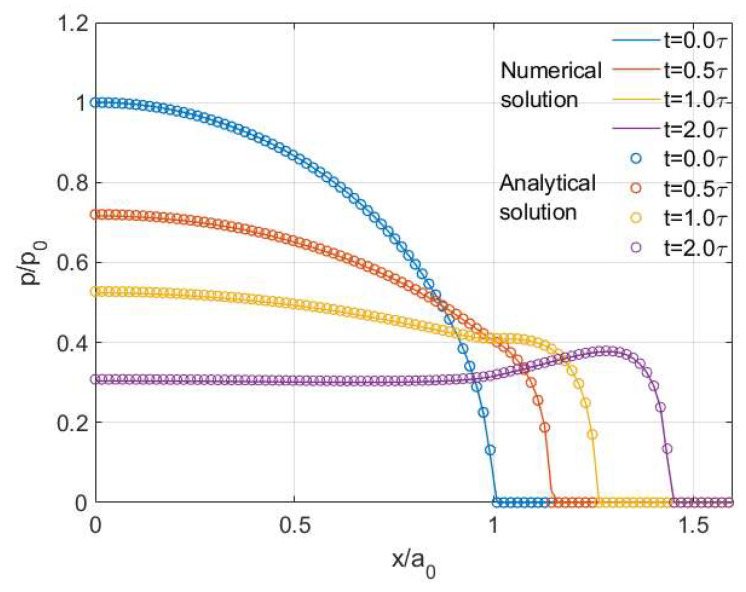
Normalized pressure *p* for the viscoelastic sphere contact (Maxwell model).

The instantaneous geometrical change of the two contacting bodies under the constant load is shown in Figure 4, where the interaction region (i.e., contacting area) is observed to keep increasing with time. This growing area tends not to diminish over time as the Maxwell model has no so-called steady state and thus cannot fully characterize the creep phenomenon. The normalized pressure distribution at different times during the viscoelastic contact process is shown in Figure 5, where the peak pressure keeps decreasing as the contacting area increases with time. Under constant normal load, the position of peak pressure is found to change with time and a spike on the contacting edge is observed after a relatively long time (t=2.0τ). To validate the simulation results, the numerical solutions (normal pressure distribution p(t)) are compared with Lee and Radok’s [3] analytical solutions expressed as follows:

(24) $$p\left(r,t\right)=\frac{2}{\pi R\left(1-\nu \right)}\int_{0}^{t}\Psi \left(t-t^{\prime }\right)\frac{d}{dt^{\prime }}\left(Re\sqrt{a^{2}\left(t^{\prime }\right)-r^{2}\;}\right)\;dt^{\prime }$$

where the operator ‘Re ()’ denotes taking the real part of the complex quantity and the time-dependent contact radius a(t) is determined as follows [3]:

(25) $$a^{3}\left(t\right)=\frac{3\left(1-\nu \right)}{4}RW\Phi \left(t\right)$$

A good agreement between the analytical solutions (scatter plots) and simulation results (solid lines) can be found in Figure 5.

To account for an arbitrary history of loading conditions, the contact of a rigid sphere (R=3.5 mm) indenting a real viscoelastic material exhibiting more than one relaxation time under dynamic loading conditions was considered. The contact behaviour of a thermoplastic polymer known as polymethyl methacrylate (PMMA), which satisfies the assumption of linear viscoelastic material, is simulated under a triangle-shaped loading condition expressed as follows:

(26) W(t)=10t·W(t)−20(t−10)·H(t−10), (N)

where H(t) denotes the Heaviside step function. 

Based on the results of a relevant standard relaxation test conducted by Kumar and Narasimhan [38], a generalized Weichert model was established by Chen et al. [11] to characterize the mechanical response of PMMA. The relaxation modulus is indicated by a two-term Prony series (two relaxation times):

(27) $$\left(t\right)=1429.71+184.62\cdot exp\left(-\frac{t}{8.93}\right)+191.06\cdot exp\left(-\frac{t}{117.96}\right),\left(MPa\right)$$

The creep compliance function for PMMA is then determined based on the relation between the relaxation modulus and creep compliance of linear viscoelastic materials in the Laplace domain (Equation (6)):

(28) $$\Phi \left(t\right)=0.699-0.0838\cdot exp\left(-\frac{t}{133.869}\right)-0.06174\cdot exp\left(-\frac{t}{9.9404}\right),\;\left(\frac{1}{GPa}\right)$$

A time-independent Poisson’s ratio (ν=0.38) is assumed here referring to Chen et al.’s work [11]. The area of interest (1280 μm×1280 μm) is discretized by a mesh system with 256×256 uniformly distributed elements, and the whole viscoelastic contact period (T=20 s) is subdivided into 41 time steps. For validation reasons, the coupling between the normal pressures and shear tractions derived from the dissimilar material properties of the PMMA half-space and the rigid plane is neglected.

**Figure 6:**
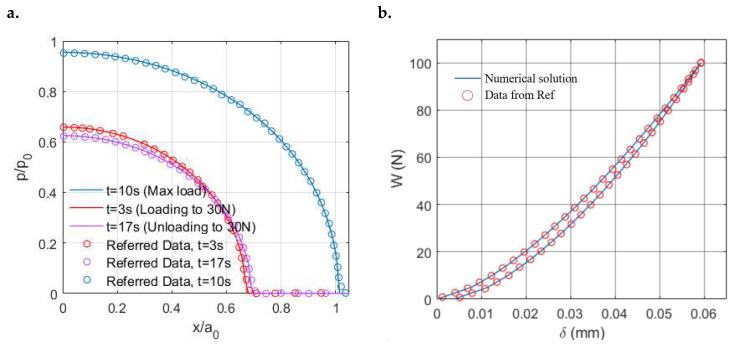
Comparison of the contact solutions from the present model (solid lines) with the results from Chen et al. [11] (scatter) under the triangular-shaped loading conditions: (**a**) Normalized pressure *p* at different times and (**b**) Evolution of rigid body indentation δ with the varying normal load W.

The pressure distributions at different time points are presented in Figure 6a, which are normalized by the Hertzian solution based on the instantaneous modulus Ψ(0) and the maximum input load $W_{max}$. The pressures at the loading period and unloading period are found to be different even though the magnitudes of the indentation load are identical. The pressure at the unloading period has a lower peak value but a wider covering range indicating a more conforming contact. Such a difference shows the effects of the loading history on the solutions to viscoelastic contact problems. Good agreement can be found between our simulation results and those derived from Chen et al.’s model [11], as shown in Figure 6a. 

Another validation in terms of the evolution of the rigid body indentation with the varying indentation load is given in Figure 6b, where the simulation results from our model agree with the referred solution of Chen et al. [11]. The remaining indentation corresponds to the unrecovered surface deformation arising from the viscosity of PMMA. The out-of-phase response of the displacement to the input load is known as hysteresis, which results from the strain energy lost or dissipated as heat between the loading and unloading periods due to internal friction in the viscoelastic material. To describe it in detail, it arises from the viscous component of the material properties. When a force is applied to a viscoelastic material, there exists a certain resistance to this input force leading to more energy (larger area under the loading curve) to be exerted than would have been expected to extend a similar purely elastic material. Such a resistance still performs during the unloading period leading to a lower amount of energy returned (smaller area under the unloading curve). Owing to this specific capacity to dissipate energy, viscoelastic materials are commonly used to produce protective products in practice.

## 4. Results and Discussion

The validated model is employed to simulate how the separation of stick and slip regions within the contacting area evolves in a viscoelastic frictional contact problem. Based on a material parameter that characterizes the rheological property of materials, a new unified explanation of the viscoelastic contact behaviour under different conditions is provided here. These simulation results are somehow counterintuitive from the perspective of the traditional theory of elastic solid contact.

Before the stick–slip simulation study, we want to raise an argument regarding an intrinsic behaviour affecting the normal pressure distribution in viscoelastic contacts.

### 4.1. What Affects the Shape of Pressure Distribution?

**Figure 7:**
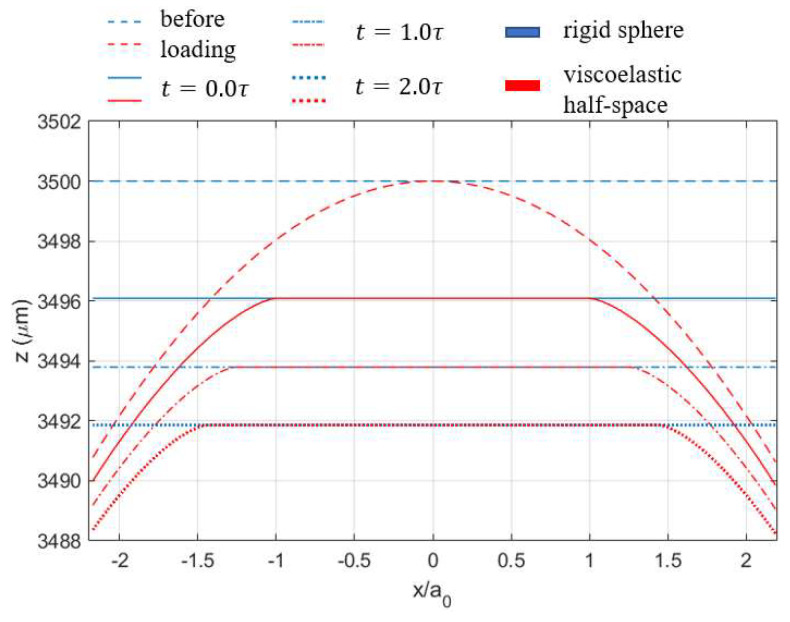
Geometry changes in the surface profiles at different times for the contact problem of a rigid plane indenting a viscoelastic sphere (Maxwell model) under constant normal load.

In a few studies concerning viscoelastic contact analysis [3,39], the spikes appearing on the edges in the normal pressure distribution, as shown in Figure 5, were argued to be one of the features of viscoelastic materials. Such an opinion is not rigorously validated since the Hertzian-type contact pressure was reported by Spinu and Cerlinca [40], whereas the spikes on the edges were found by Bugnicourt [39]. Both studies used Zener models to characterize the viscoelastic response of the simulated materials in their studies. It was also proposed by Koumi et al. [21] that the configuration of the two contacting bodies determines the shape of the pressure distribution. In other words, the shape of the contact tractions depends on which one of the two contacting bodies is viscoelastic, whereas the other could be considered rigid. According to Koumi et al. [21], when it comes to the contact between a sphere and a half-space, if the sphere is rigid and the half-space is viscoelastic, the normal pressure will contain spikes on the edges. Contrarily, the Hertzian-type contact pressure is obtained. However, the authors of the current manuscript have tested both cases using a Maxwell model with the properties given in Table 1. The spikes on the contacting edges appear in the normal pressure profiles after a relatively long time (t≥1.0τ) in both cases, which contradicts the claims of Koumi et al. [21]. The solutions to the surface displacement and normal pressure are identical to that presented in Figure 5 no matter which one of the contacting bodies is viscoelastic. Compared with the solutions shown in Section 3 using the Maxwell model, the only dissimilarity arising from an opposite contact configuration is the geometry change in the two contacting surfaces at different time points, as shown in Figure 7. In this case, identical magnitudes of deformation and indentation are now experienced by the sphere and flat rigid plane, respectively. It is of note that in Koumi et al.’s study [21], the normal displacement is specified, whereas the indentation load is specified in the current study.

Considering the limitation of Maxwell models to quantify the viscoelastic response of materials, several simulation attempts using Zener models were conducted. The relaxation modulus and creep compliance of a Zener model are usually defined in the following general form in several numerical studies [18,19,20,21,22,23]:

(29) $$\Psi \left(t\right)=\mu_{0}+\left(\mu_{\;\infty }-\mu_{0}\right)exp\;\left(-\frac{t}{\frac{\mu_{0}}{\mu_{\infty }}\tau }\right),$$

(30) $$\begin{array}{c} \Phi \left(t\right)=\frac{1}{\mu_{\infty }}+\frac{1}{\mu_{1}}\left(1-exp\left(-\frac{t}{\tau }\right)\right),\\ \mu_{1}=\frac{1}{\frac{1}{\mu_{0}}-\frac{1}{\mu_{\infty }}}, \end{array}$$

where $\mu_{\infty }$ is the initial shear modulus, $\mu_{0}$ is the modulus after infinite time; the ratio $\left(\mu_{\infty }/\mu_{0}\right)$ is known as the ratio of the retardation time (characteristic time for creep) to the relaxation time and it was commonly specified to be 10 in different numerical simulations of viscoelastic contact problems conducted by many researchers [18,21,22,23]. 

To investigate the physical meaning of the ratio ($\mu_{\infty }/\mu_{0}$) and show its effects on the contact solutions, some of the material properties including the instantaneous modulus $\mu_{\infty }$, the relaxation time τ, the Poisson’s ratio ν, the geometry of the contacting bodies, and the indentation load were set to be identical to those in the study by Wallace [22]. In addition, the ratio ($\mu_{\infty }/\mu_{0}$) is specified to vary within a certain range, as shown in Table 2. The potential contact area ($5.5a_{0}\times 5.5a_{0}$) is discretized by a mesh system with 256×256 uniformly distributed elements, and the whole viscoelastic contact period (T=3τ) is subdivided into a number of time points depending on the ratio value.

**Figure 8:**
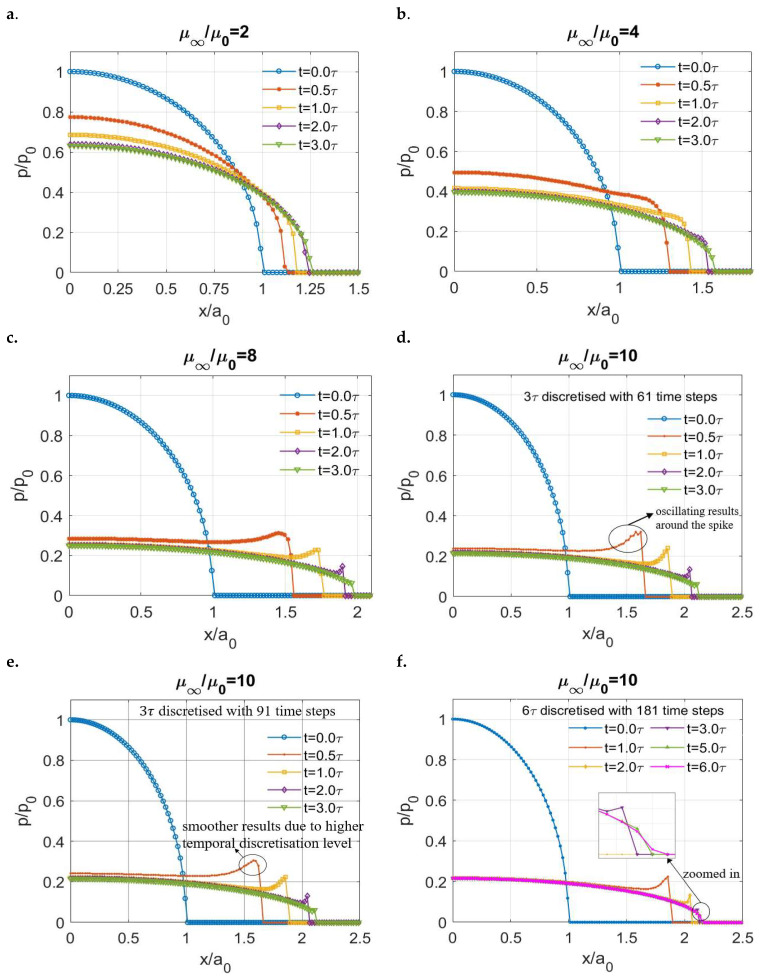
Normalized normal pressure distribution at different moments for different viscoelastic materials (Zener model) within the simulation time: (**a**) $\frac{\mu_{\infty }}{\mu_{0}}=2$, (**b**)$\;\frac{\mu_{\infty }}{\mu_{0}}=4$, (**c**)$\;\frac{\mu_{\infty }}{\mu_{0}}=8$, (**d**) $\frac{\mu_{\infty }}{\mu_{0}}=10$, (**e**)$\;\frac{\mu_{\infty }}{\mu_{0}}=10$ with more time steps and (**f**) $\frac{\mu_{\infty }}{\mu_{0}}=10$ with extended simulation time.

The normalized results presented in Figure 8a–e show that the shape of normal pressure in a viscoelastic indentation problem is significantly affected by the ratio ($\mu_{\infty }/\mu_{0}$) of a material. The spikes on the contacting edge are only observed for viscoelastic materials with a relatively high ratio (Figure 8c for $\mu_{\infty }/\mu_{0}=8$ and Figure 8e for $\mu_{\infty }/\mu_{0}$ = 10), whereas a Hertzian-type normal pressure distribution is observed for the materials with a lower ratio (Figure 8a for $\mu_{\infty }/\mu_{0}=2$ and Figure 8b for $\mu_{\infty }/\mu_{0}$ = 4). It is of note that for the simulation of viscoelastic materials with higher ratios of $\left(\mu_{\infty }/\mu_{0}\right)$, more time steps are usually required to discretize the specified time domain to avoid the oscillating results around the contacting edge during the early period of simulation, as labelled in Figure 8d. The distinct shapes of the pressure distribution for viscoelastic materials characterized by standard linear solids with different ratios $\left(\mu_{\infty }/\mu_{0}\right)$ were also reported by Yakovanko and Goryacheva [41], but there exists no detailed interpretation of the outcome. 

The spikes on the contacting edges do not exist permanently. After extending the simulation time from 3τ to 6τ for the contact simulation of the viscoelastic material with a ratio $\mu_{\infty }/\mu_{0}$ = 10, the spikes disappear and the pressure distribution becomes the Hertzian-type at the end of the simulation time as shown in Figure 8f. In addition, the solutions are found to be close to each other after a relatively long time (t≥5.0τ), which implies that a steady state is almost reached for the viscoelastic contact. 

**Figure 9:**
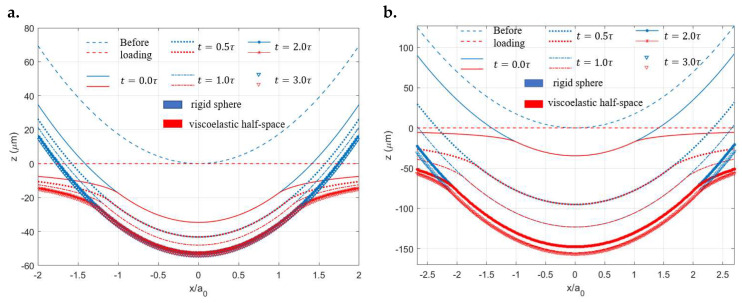
Creep of surface profiles at different time points for different viscoelastic materials: (**a**) $\frac{\mu_{\infty }}{\mu_{0}}=2$ and (**b**) $\frac{\mu_{\infty }}{\mu_{0}}=10$.

To explain the observed phenomena, the changes in surface geometry for the materials with the lowest and highest ratios of $\mu_{\infty }/\mu_{0}$ under the constant indentation load within the same simulation time (Figure 9a,b, respectively) are compared. The viscoelastic surface with the higher ratio of $\mu_{\infty }/\mu_{0}$ experiences dramatically increasing deformation with time, during which the contact becomes significantly more conformal. In other words, the creep phenomenon is more intense for the surface with a higher ratio. To assume that a viscoelastic body experiences such a weak creep that the change in surface deformation per time is extremely insignificant, it could be approximated as an ordinary elastic solid. One exhibiting a converse contact behaviour could then be treated as a fluid. Based on the assumption, the ratio of the retardation time to relaxation time determines whether a viscoelastic is more fluid-like or solid-like. For materials with lower ratios of $\mu_{\infty }/\mu_{0}$, the elasticity plays a dominant role leading to a Hertzian-type pressure distribution. In this case, the viscosity of the materials has relatively trivial effects causing the increase in the contacting area with a rapidly decreasing strain rate. Eventually, the steady state of the viscoelastic contact is quickly reached. For materials with higher ratios of $\mu_{\infty }/\mu_{0}$, their responses to the indentation load are similar to that of a pack of liquid once squeezed. Under this condition, the load is distributed to the contacting edges when the material is squeezed after a period of time. However, due to a decreasing strain rate, a steady state would be reached for any viscoelastic contact using a Zener model. The pressure distribution eventually evolves into the Hertzian type arising from the elasticity of materials. The time needed to reach this state is also determined by the ratio value so that a higher ratio leads to a longer time.

It is of note that for viscoelastic solids, the retardation time must always be greater than the relaxation time, which means that the ratio $\mu_{\infty }/\mu_{0}$ should always be more than 1. A ratio that is less than 1 is normally valid for viscoelastic fluids that can be characterized with an anti-Zener model [42].

**Figure 10:**
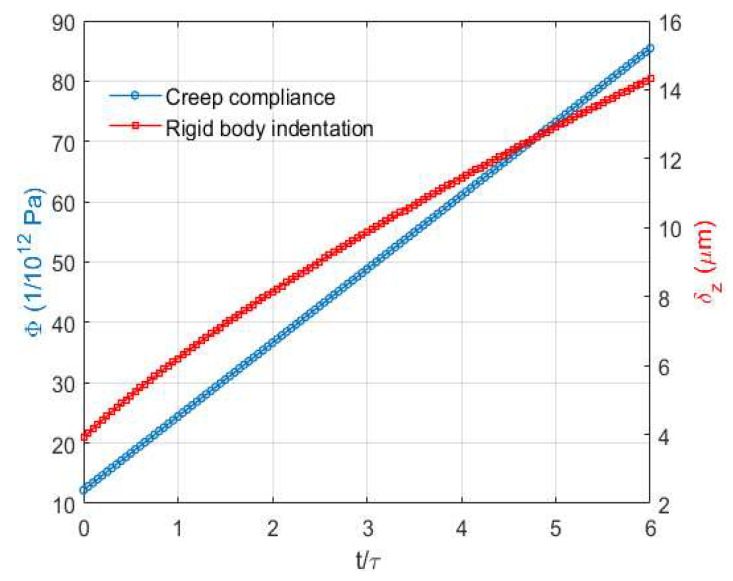
Changes in the creep compliance Φ (y axis right) and the resulting rigid body indentation $\delta_{z}$ (y axis right) with time.

**Figure 11:**
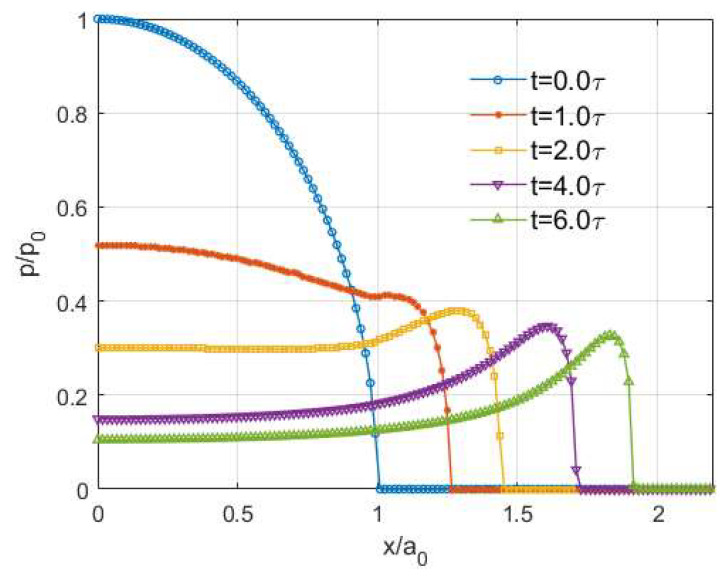
Distribution of normal pressure at different time points within the simulation window (t=6.0τ).

Due to the linearly increasing creep compliance, the Maxwell model shows no decreasing strain rate as shown in Figure 10. According to Chen et al. [11], a Maxwell model is usually employed to characterize the evident viscoelastic response exhibited by soft thermoplastic polymers in the vicinity of their melting temperature. Even though the simulation time for the contact problem of the Maxwell model investigated in Section 3 is extended, the normalized results (Figure 11) show that the spikes on the edges always exist for the normal pressure distribution. This indicates that the load keeps being distributed on the contact edges because of its extremely strong fluid-like properties. 

For viscoelastic materials characterized by the generalized Weichert models exhibiting more than one relaxation time, there exist multiple ratios of $\mu_{\infty }/\mu_{0}$. To find a general rule in this case, the normal indentation problems of PMMA and a fictitious viscoelastic material were simulated. The Poisson’s ratio of the fictitious material is the same as PMMA (ν=0.38) but the creep compliance is adjusted based on that of PMMA (Equation (28)) as follows:

(31) $$\Phi \left(t\right)=0.699-0.5595\cdot \exp\left(-\frac{t}{133.869}\right)-0.02\cdot exp\left(-\frac{t}{9.9404}\right),\;\left(\frac{1}{\mathrm{GPa}}\right)$$

The two ratios $\left(\mu_{\infty }/\mu_{0}\right)$ of the materials characterized by a two-element generalized Weichert model are evaluated approximately by transforming the creep compliance function into the forms in two Zener models. To take the PMMA material as an example, its creep compliance (Equation (28)) is converted into the following forms:

(32) $$\Phi {\left(t\right)}_{1}=0.699-0.0838\cdot \exp\left(-\frac{t}{133.869}\right),\;\left(\frac{1}{\mathrm{GPa}}\right)$$

(33) $$\Phi {\left(t\right)}_{2}=0.699-0.06174\cdot exp\;\left(-\frac{t}{9.9404}\right),\;\left(\frac{1}{\mathrm{GPa}}\right)$$

which leads to two relatively low ratios ($\mu_{\infty }/\mu_{0}{}_{1}\approx 1.136$ and $\mu_{\infty }/\mu_{0}{}_{2}\approx 1.097$). Using the same method, the ratios of the fictitious viscoelastic material are determined as well ($\mu_{\infty }/\mu_{0}{}_{1}\approx 5$ and $\mu_{\infty }/\mu_{0}{}_{2}\approx 1.03$).

The surface geometry, contact configuration, and spatial discretization used for the indentation simulation are identical to that specified for the dynamic loading contact problem of PMMA in Section 3. A step load of 10 N is specified at the beginning of the simulation and held constant for 600 s, which is discretized into 61 and 121 time steps for the contacts of PMMA and fictitious material, respectively. 

**Figure 12:**
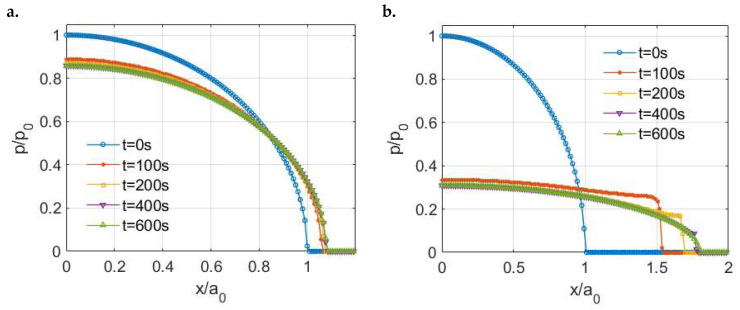
Pressure distributions for different viscoelastic materials (generalized Weichert model) under normal loading: (**a**) PMMA and (**b**) Fictitious viscoelastic material.

Under these contact inputs, the PMMA is found to experience such relatively insignificant creep deformations that the steady-state contact is quickly reached as shown in Figure 12a. Since the two ratios of PMMA are extremely small, the PMMA surface should perform more like a solid. On the contrary, spikes on the edges could be observed in the normalized pressure profiles of the fictitious material (Figure 12b) although a Hertzian-type pressure appears after a relatively long simulation time. A short-term conclusion regarding the generalized Weichert model can be drawn so that as long as one of the ratios is high enough that the material can be considered more fluid-like, spikes on the edges would be observed for the normal pressure distribution at a limited period. 

### 4.2. Does the Viscoelasticity Affect the Stick and Slip Separation?

#### 4.2.1. Stick–Slip under Constant Inputs

A normal load alone can result in the partial slip contact of two dissimilar materials when the coupling between normal pressure and shear tractions is considered. The study of viscoelastic partial slip contact can be performed without any tangential load. Based on our understanding of the effects of material ratio ($\mu_{\infty }/\mu_{0}$) on the contact solutions, Zener models with two different ratio values ($\mu_{\infty }/\mu_{0}{}_{1}=3$ and $\mu_{\infty }/\mu_{0}{}_{2}=10$) are employed here to simulate the partial slip contacts of different viscoelastic materials. Some of the contact inputs including the surface geometry, contact configuration, spatial discretization, and normal load are specified, as those given in Table 2, whereas the total simulation time (T=2τ) is discretized with 81 time steps. A constant coefficient of friction $\mu_{f}$ is assumed to be 0.30.

**Figure 13:**
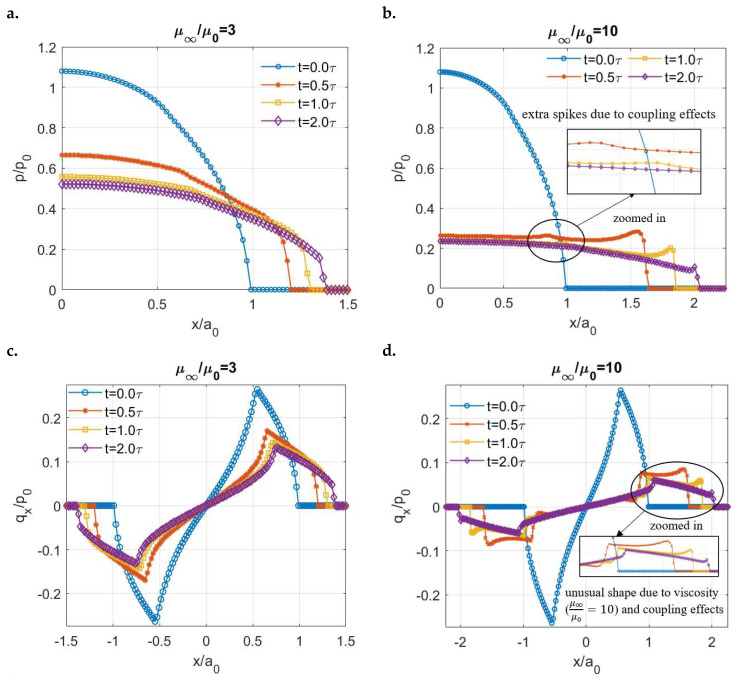
Coupled contact solutions of different viscoelastic materials under normal load alone: (**a**,**b**): Normalized pressure profiles for $\frac{\mu_{\infty }}{\mu_{0}}=3$ and $\frac{\mu_{\infty }}{\mu_{0}}=10$ respectively and (**c**,**d**): Normalized shear tractions for $\frac{\mu_{\infty }}{\mu_{0}}=3$ and $\frac{\mu_{\infty }}{\mu_{0}}=10$ respectively.

The normalized contact solutions for these two different viscoelastic materials are given in Figure 13. Since a tangential load has not been applied yet, the normal pressure distributions of the two surfaces still exhibit axial symmetry. For simplicity, only the pressures on the front part of the contacting areas are presented in Figure 13a,b, where the peak pressure at the beginning is higher than the Hertzian pressure due to the coupling effects. The normal pressure profile of the viscoelastic surface with the smaller ratio shows no significant change related to the coupling effects. In comparison, the extra spikes in the middle of the contacting region, as indicated in Figure 13b, are found for the viscoelastic surface with a higher ratio due to the coupling. To explain such results in detail, the solutions of the normal pressure and shear tractions are interdependent in a coupled case. For the more solid-like viscoelastic surface $\left(\mu_{\infty }/\mu_{0}=3\right)$, a Hertzian-type pressure distribution is expected in the uncoupled case arising from the dominant performance of its elasticity. The resulting shear tractions are expected to be similar to those that could be achieved from a purely elastic surface. Although its peak value decreases as the contacting area increases with time, the shear traction results of the more solid-like material (Figure 13c) agree with this hypothesis. For the more fluid-like viscoelastic surface $\left(\mu_{\infty }/\mu_{0}=10\right)$, pressure spikes on the contacting edges are expected before the steady-state time point under uncoupled conditions. As a result, additional irregular features appear on its shear traction profile, as highlighted in Figure 13d, when the coupling effects are included. These irregularities in turn affect the pressure distribution leading to those extra spikes.

**Figure 14:**
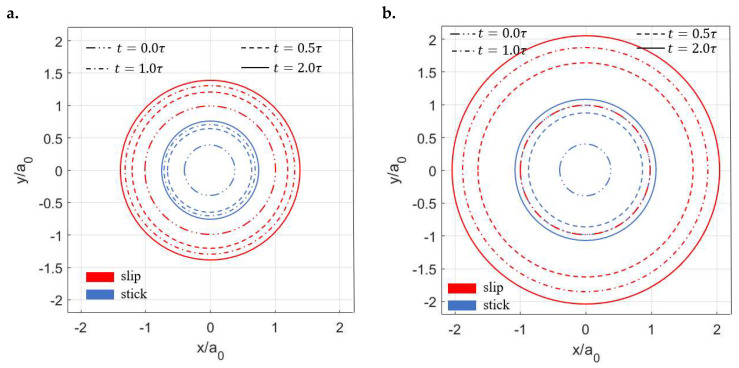
Separation of stick and slip regions with time for different viscoelastic materials: (**a**) $\frac{\mu_{\infty }}{\mu_{0}}=3$ and (**b**) $\frac{\mu_{\infty }}{\mu_{0}}=10$ (the blue lines are the boundaries of the stick regions and the regions between the blue lines and red lines are the slip regions).

**Figure 15:**
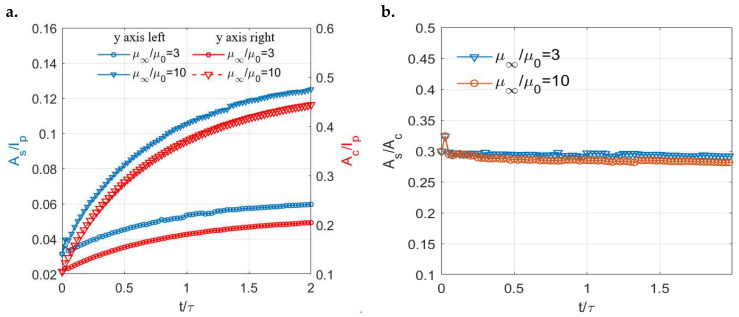
Simulation solutions of different viscoelastic materials under normal load alone: (**a**) Variations in the ratios of the stick region (*A_s_*/*I_p_*, left *y* axis) and contacting region to the simulation domain (*A_c_*/*I_p_*, right *y* axis) with time for different viscoelastic materials and (**b**) Variations in the ratios of the stick region to the contacting region (short for stick ratio: *A_s_*/*A_c_*) with time for different viscoelastic materials.

Despite the distinct profiles of the contact tractions, the stick–slip separations of the two viscoelastic surfaces show similar shapes as shown in Figure 14. In both cases, the contacting area is separated into a central stick region (region surrounded by the blue line) and a surrounding annulus (the region between the red line and blue line) regardless of the time. The areas of both the stick $\left(A_{s}\right)$ and contacting zones ($A_{c}$) are found to increase with the ratios $\left(\mu_{\infty }/\mu_{0}\right)$ of the viscoelastic materials (Figure 15a). However, as illustrated in Figure 15b, the ratios of the stick region to the contacting region (stick ratio hereinafter) do not vary much between the two investigated viscoelastic materials. This suggests that the stick ratio is insensitive to the viscoelasticity of materials under the current loading conditions.

To further investigate the stick–slip contact for viscoelastic materials under constant inputs, a Zener model with an intermediate ratio value ($\mu_{\infty }/\mu_{0}=4$) is employed. It is of note that the solutions to viscoelastic contact problems depend on the way the contact is imposed. To visualize the specific features of viscoelastic materials, two separate cases are first studied here: constant load and constant displacement in the lateral direction. Most of the simulation inputs remain the same as in the former case (no input in the lateral direction), except that the input in the x direction is now changed to be a constant load ($F_{x}=0.5\mu_{f}W$) or a constant displacement $\left(\delta_{x}=6.1987\right)$. In other words, two different contact phenomena in the x direction, including creep and stress relaxation, are simulated here. It is of note that a load-controlled algorithm in the tangential direction could fail to converge when the contact of a viscoelastic material with a relatively high ratio is considered. A flow chart showing the search method for contact solutions in the tangential direction is given in the Appendix A. In the algorithm, the convergence speed of the outer loop regarding the load balance relies heavily on how the updated shear tractions adjust the tangential displacements ($\delta_{x}$ and $\delta_{y}$) to make them close to the correct solutions. When it comes to the contact of a more fluid-like viscoelastic material, the incremental deformation derived from the previous tractions $u_{incre}$ determined from Equation (19) could be large after a relatively long time. Therefore, the contribution of the deformation derived from the shear traction at the current time step $u_{t}$ determined from Equation (21) does not significantly affect the total deformation. As a result, the changes in the current time-step shear tractions fail to search for the right tangential displacement, which results in an endless loop regarding the load balance in the tangential direction. To obtain the partial slip solution of a viscoelastic surface under constant loading conditions, based on an extrapolation approach, a dynamic rigid body displacement in the x direction is specified in the displacement-controlled algorithm to obtain results with a constant tangential loading. The partial slip simulation test under this input condition is named the load-constant (LC) test to facilitate the following description and the one under the input of constant displacement in the x direction is named the displacement-constant (DC) test.

**Figure 16:**
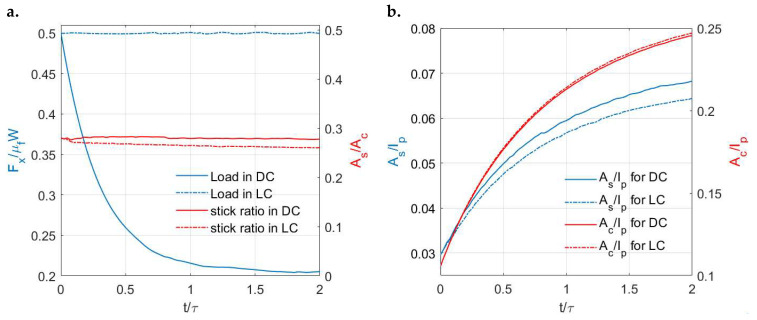
Simulation solutions of viscoelastic partial slip contact under different input conditions: (**a**) Variations in the tangential loads (*y* axis left) and stick ratios (*y* axis right) with time in the DC and LC cases and (**b**) Variations in the ratio of the stick region to the simulation domain (*y* axis left) and the ratio of the contacting area to the simulation domain with time (*y* axis right) in the DC and LC cases.

The variations in the tangential load with time in these two cases are shown in Figure 16a. The load is kept constant for the LC case (creep), whereas the stress relaxation phenomenon is simulated for the DC case. Due to the relaxed load in the DC case, its stick ratio is lower than that in the LC case after a certain time but the difference tends to be insignificant. Figure 16b shows the variations in the contacting area and stick areas with time under the two different input systems. The growth rate of the stick region in the DC case is higher under the effects of stress relaxation. Although a constant normal load is applied in both tests, the growth rate of the contacting area in the LC case is higher. To explain this, apart from the contribution of surface creep under normal loading, the higher tangential load in the LC case leads to slight additional contacting areas under the coupling effects of each time step.

**Figure 17:**
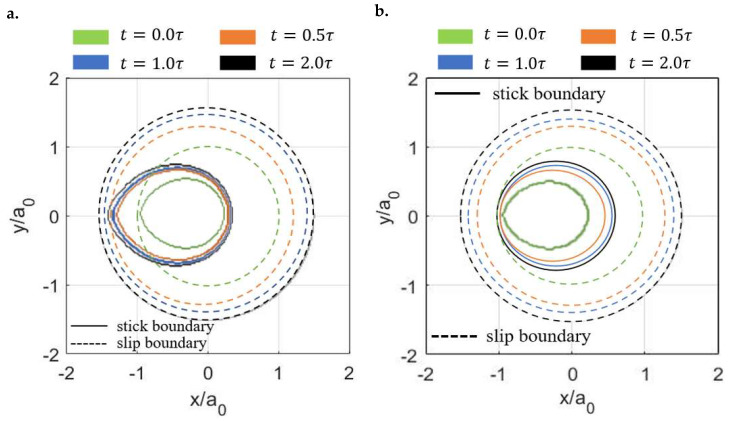
Separations of the stick and slip regions under constant inputs in *x* directions: (**a**) LC case and (**b**) DC case (the solid lines are the boundaries of the stick regions and the regions between the solid lines and dashed lines are the slip regions).

**Figure 18:**
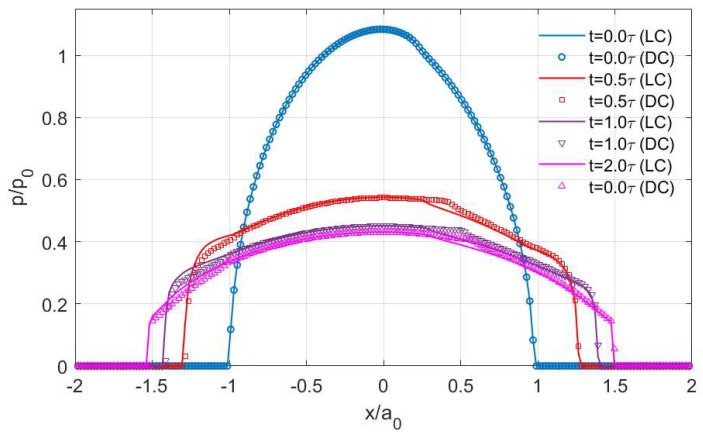
Changes in normal pressure distribution *p* along the *x* axis with time in the LC (solid line) and DC (scatter plot) cases.

**Figure 19:**
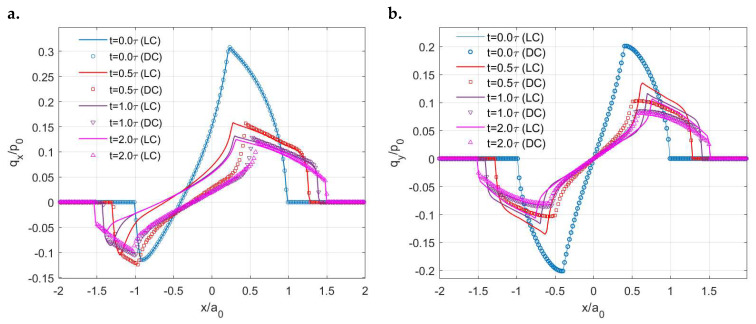
Changes in shear traction distribution with time in the LC (solid line) and DC (scatter plot) cases: (**a**) *q_x_* along the *x* axis and (**b**) *q_y_* along the *y* axis.

The creep (LC) and stress relaxation (DC) encountered in the *x* direction result in different separation patterns of stick and slip regions with time. As shown in Figure 17, the shape of the stick regions in the LC case does not change with time (always a raindrop shape), whereas that in the DC case varies (from a raindrop shape to a circular shape). Furthermore, the position of the stick region changes with time in the DC case, which starts from almost the rearrest zone of the contacting area and then shifts to the front. Such a separation of stick and slip regions is expected according to the distribution of the contact tractions. The normal pressures are shown in Figure 18. Compared to the solutions in the LC case, it is evident that the DC case shows a higher peak pressure and its position is closer to the front part of the contacting area. Also, the pressures on the rear section of the contacting area are found to be lower in the DC case, whereas those on the front section are higher. Figure 19a,b show the normalized shear tractions ($q_{x}$ and $q_{y}$, respectively) for the two cases, where the DC case tends to show higher $q_{x}$ at the rear zone of the contacting area and lower $q_{x}$ at the front zone. The area in which there is relatively low normal pressure or high shear traction is more prone to slip as it is easier for the shear tractions to reach the boundary of local friction following Coulomb’s law. As a result, there exist more slip regions at the rear part of contacting area in the DC case.

**Figure 20:**
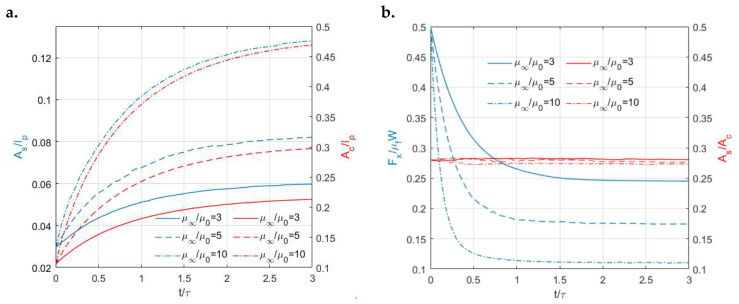
Solutions to the stick–slip contact of different viscoelastic materials (Zener model) under constant displacement in *x* direction: (**a**) Variations in the ratio of the stick region to the simulation domain (*y* axis left) and the ratio of the contacting area to the simulation domain (*y* axis right) with time and (**b**) Variations in the resulting tangential loads (*y* axis left) and stick ratios (*y* axis right) with time for different viscoelastic materials (Zener model).

To study the effects of the material properties $\left(\mu_{\infty }/\mu_{0}\right)$ on the stick and slip separation within the contacting area under constant displacement input in the tangential direction (DC condition), Zener models with three different ratio values ($\mu_{\infty }/\mu_{0}{}_{1}=$3, $\mu_{\infty }/\mu_{0}{}_{2}=$5, $\mu_{\infty }/\mu_{0}{}_{3}=$10) were employed. As expected, different growth rates of stick and contacting areas are experienced by these materials, as shown in Figure 20a. A lower stick ratio is observed in Figure 20b for the viscoelastic material with a higher ratio $\left(\mu_{\infty }/\mu_{0}\right)$. However, the differences between the stick ratios of the three cases are not significant despite a more notable stress relaxation phenomenon exhibited by the material with a higher ratio.

**Figure 21:**
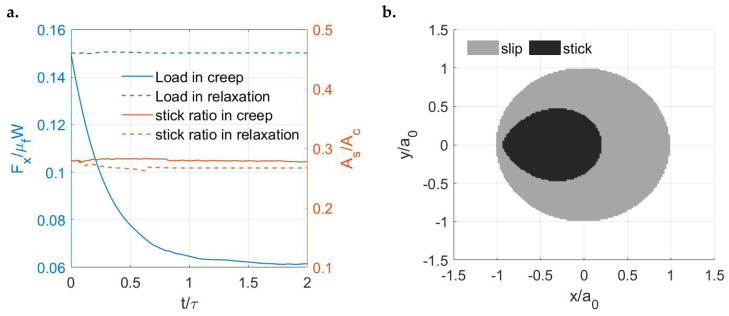
(**a**) Variations in the resulting tangential loads (*y* axis left) and stick ratios (*y* axis right) with time in the relaxation (solid line) and creep (scatter) cases and (**b**) Time-independent separation pattern of the stick (dark) and slip (grey) regions in the relaxation case.

**Figure 22:**
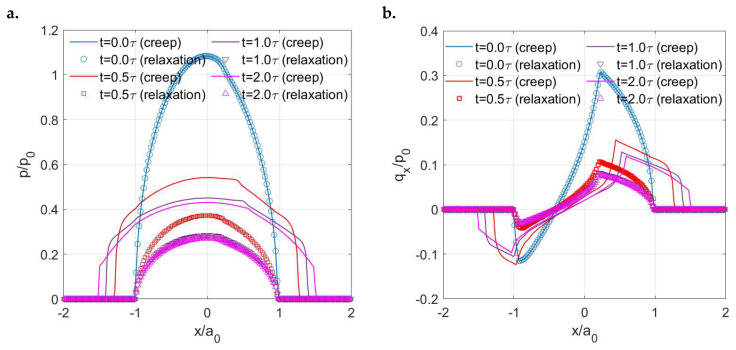
Changes in the contact tractions with time for the creep (solid line) and relaxation (scatter) cases: (**a**) pressure *p* along the *x* axis and (**b**) shear traction *q_x_* along the *x* axis.

Now, to vary the contact input in the normal direction, the Zener model ($\mu_{\infty }/\mu_{0}=4$) is tested again but with constant displacement in both the normal and tangential directions ($\delta_{z}=34.358$ and $\delta_{x}=6.1987$). In other words, the stress relaxation phenomenon is encountered in both the normal and tangential directions. To facilitate subsequent clarification, the current test is named the relaxation case and the former test (DC case) with the constant displacement in the tangential direction but the constant load in the normal direction is now named the creep case. As shown in Figure 21a, the ratio of the tangential load to the static friction remains constant with time for the relaxation case, whereas it drops for the former relaxation case. This consequently leads to a slightly lower stick ratio for the relaxation case, as indicated in Figure 21a. Unlike the results of the former creep case (Figure 17b), the shape and size of the stick and slip regions for the current relaxation case are found to be time-independent, which follows a certain separation pattern, as shown in Figure 21b. Such results can be expected based on the contact tractions of the corresponding case. As shown in Figure 22, the normal pressure and shear tractions in the relaxation case vary insignificantly with time when compared to those in the former creep case. 

Combing the results of the above partial slip tests, a short-term conclusion can be drawn. The stick ratio is insensitive to the time-varying property of viscoelastic materials as long as the specified contact inputs (displacement or load) in the normal and tangential directions are constant. However, the separation pattern of the stick and slip regions depends on the contact phenomena experienced by the viscoelastic surface in the normal and tangential directions. When different contact phenomena are encountered (different types of contact inputs are specified) in the normal and tangential directions, the separation pattern of the stick and slip regions varies with time. Otherwise, it tends to be time-independent. 

#### 4.2.2. Evolution of Stick–Slip with Increasing Displacement

**Figure 23:**
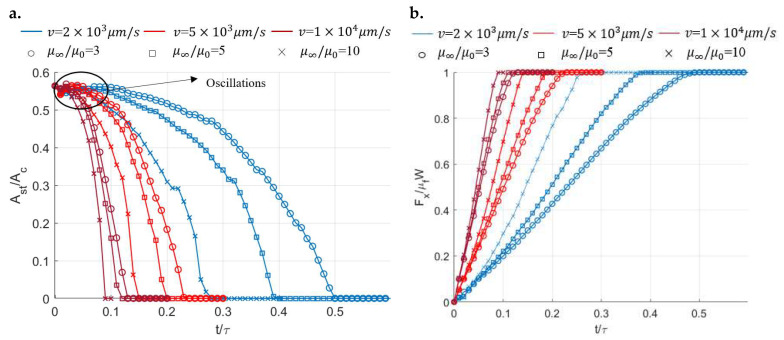
Simulation results under semi-coupled conditions: (**a**) Evolution of the stick ratio with time for different viscoelastic materials and (**b**) Evolution of the resulting tangential loads with time for different viscoelastic materials (*v* is the increasing rate of tangential displacement).

To investigate the transition from partial slip to gross sliding for different viscoelastic materials, a dynamic loading profile is specified in the lateral direction. The simulation starts with the semi-coupled condition, where only the coupling between the pressure and shear traction in the x direction is considered. The contact inputs employed here are a constant displacement in the normal direction and a linearly increasing displacement in the x direction. The surface geometry, contact configuration, coefficient of friction, spatial discretization, and input normal displacement are the same as those used in the former partial slip simulations in Section 4.2.1. By varying the ratio ($\mu_{\infty }/\mu_{0}$) of the materials and also the increasing rate of tangential displacement v in the x direction, the stick ratio with time for different materials under the different increasing rates of tangential displacement evolves, as shown in Figure 23a. Due to the discrepancy between the linear input of tangential displacement and the nonlinear mechanical response of the viscoelastic materials, oscillating results are experienced in the early simulation period, as labelled in Figure 23a. The variations in the tangential loads resulting from the specified tangential displacements are shown in Figure 23b. As expected, a higher increasing rate of tangential displacement leads to a quicker transition from partial slip to gross sliding, in which state the tangential load is equal to the static friction ($\mu_{f}W$). In addition, under the same increasing rate of displacement, the more fluid-like material ($\mu_{\infty }/\mu_{0}$ = 10) can always reach the gross sliding state within a shorter time owing to its stronger flowability. 

**Figure 24:**
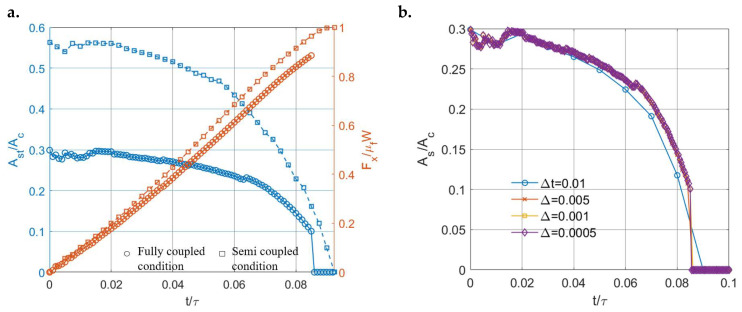
(**a**) Comparison of partial slip solutions under fully coupled (circle) and semi-coupled (square) conditions and (**b**) Comparison of partial slip solutions using different time intervals (Δ) under fully coupled conditions.

To switch to the fully coupled condition, under the same increasing rate of displacement, the tested more fluid-like material ($\frac{\mu_{\infty }}{\mu_{0}}=10$) can now reach gross sliding within a shorter time compared to the case under semi-coupled conditions, as illustrated in Figure 24a. This causes a lower tangential load required to reach gross sliding, as shown in Figure 24a. Apart from the ordinary full-coupling effects, the transition from partial slip to gross sliding now tends to be abrupt, as indicated in Figure 24a. The stick ratio jumps from a relatively high value to zero. To obtain a smoother transition, trial tests with smaller time intervals were conducted using the same contact inputs. However, the differences between those partial slip solutions are insignificant when the time intervals used are considerably smaller, as shown in Figure 24b. This suggests that the abrupt jump is inevitable when the fully coupled condition is introduced. Mathematically, such an abrupt transition can be caused by the unstable and time-varying properties of viscoelastic materials and the modified condition of gross sliding when fully coupled conditions are introduced. 

**Figure 25:**
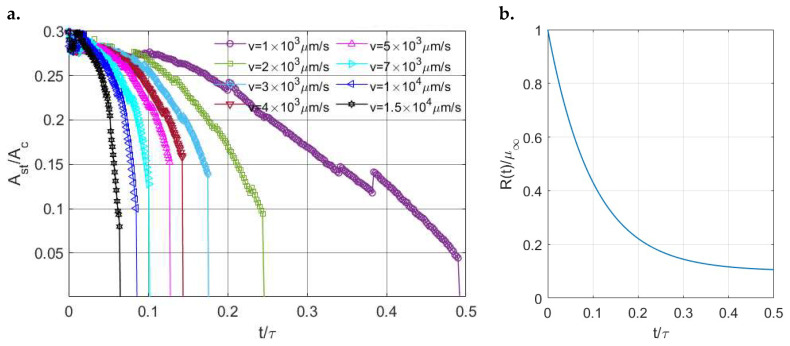
(**a**) Fully coupled solution regarding the variations in the stick ratio with time under different increasing rates of displacement and (**b**) Variations in relaxation modulus *R*(*t*) of the material with time.

To validate if such a relation holds, the fluid-like material ($\mu_{\infty }/\mu_{0}$) was further tested, for which gross sliding was obtained at different times under fully coupled conditions by varying the increasing rate of surface displacement. The results (Figure 25) show that the variations in the stick ratio just before sliding (short for critical stick ratio hereinafter) with the time when gross sliding is achieved is relevant to the evolution of the relaxation modulus of the material within the time. As shown in Figure 25a, the critical stick ratio first increases when a longer time is required for the contact to be in the sliding state. Meanwhile, the relaxation modulus drops dramatically, as shown in Figure 25b. However, the critical stick ratio starts to decrease when the changing rate of the relaxation modulus becomes lower, as indicated in Figure 25. For the purple line ($v=1\times 10^{3}$ μm/s) where gross sliding happens when the material property is steady, the transition from partial slip to gross sliding tends to be less abrupt. 

Such an abrupt transition is counterintuitive if analyzing the results within the framework of classical contact mechanics. A brand-new view concerning the rheological behaviour of viscoelastic materials is provided here to explain such results. It is well-recognized that there is a threshold concerning the tangential load needed to be reached in order to induce sliding for any elastic solid. On the other hand, fluids will flow as long as there is a shear in any lateral direction since there exists no threshold to generate relative motion. In other words, gross sliding is attained immediately when a tangential load is applied to a fluid. A viscoelastic material, as its name implies, should exhibit a response incorporating the contact characteristics of both solids and fluids. From this perspective, our coupled model shows a reasonable outcome resulting from such integrated contact behaviour. What can be observed from our results is that during the early period of transition from partial slip to gross sliding, viscoelastic surfaces tend to require the tangential displacement or the equivalent load to reach a threshold value. This is identical to the elastic case. However, the final transition tends to be an abrupt process, which is expected for a fluid, rather than a smooth one that is commonly encountered when addressing an analogous elastic stick–slip problem under a monotonically increasing tangential load or displacement. 

**Figure 26:**
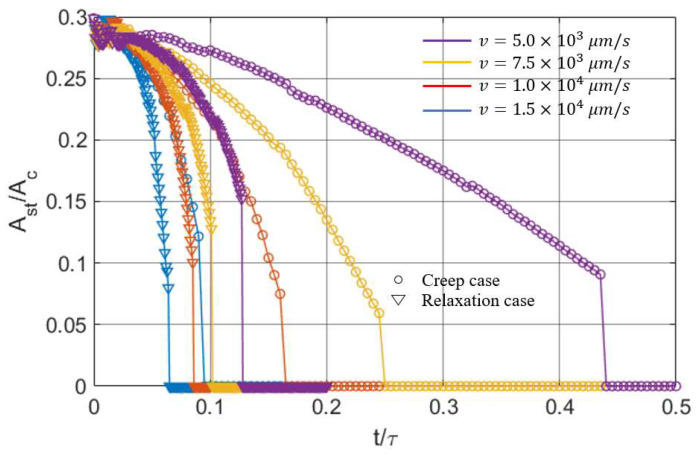
Fully coupled solution regarding the variations in the stick ratio with time under different inputs (creep or stress relaxation) in the normal direction.

**Figure 27:**
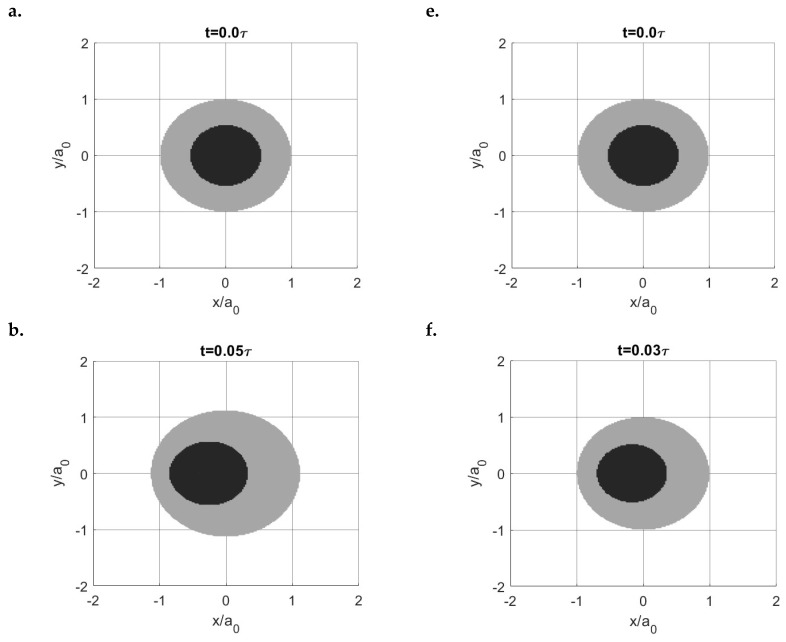

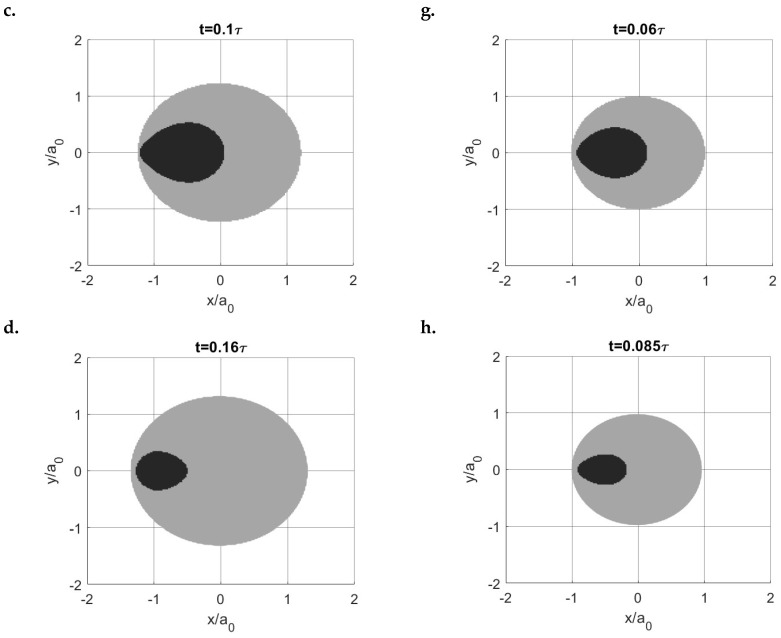
Fully coupled solution regarding the evolution of the stick (dark) and slip (grey) regions for the creep case (**a**–**d**) and relaxation case (**e**–**h**).

The partial slip problems discussed above were all simulated when a fixed displacement was specified in the normal direction. To build up the complication, the transition from partial slip to gross sliding for the fluid-like material ($\frac{\mu_{\infty }}{\mu_{0}}=10$) was simulated again but with a constant normal load. Compared to the current case where a creep phenomenon is encountered in the normal direction, the results (Figure 26) show that it is always quicker for the former stress relaxation case to reach gross sliding under an increasing rate of tangential displacement. Since the load keeps decreasing with time when a stress relaxation is experienced, the sliding condition is easier to be satisfied. Arising from such a difference, two different evolution forms of stick and slip regions within different times are obtained for the creep and relaxation cases, as shown in Figure 27.

## 5. Concluding Remarks

The three main contact phenomena of viscoelastic materials including creep, stress relaxation, and hysteresis are reproduced using the newly developed viscoelastic contact model. The ratio of retardation time to relaxation time $\left(\frac{\mu_{\infty }}{\mu_{0}}\right)$ is employed to characterize the rheological property of viscoelastic materials. Although the exact boundary value is not known, materials with high ratios are observed to exhibit more fluid-like contact behaviours, whereas those with low ratios behave more like a solid. No matter what rheological model is employed to characterize the viscoelastic material, it is the ratio $\left(\mu_{\infty }/\mu_{0}\right)$ that determines the shape of the contact tractions. Concerning the stick–slip contact analysis of different viscoelastic materials, the following conclusions can be drawn:

- For the creep contact of a more fluid-like viscoelastic material, the spike on the edge of the normal pressure distribution is observed before the steady state, which results in irregular shapes of shear tractions due to the coupling between the pressure and shear stress. The pressure spikes also influence the subsurface stress. The position of the peak stress can be shifted to the edge of the contacting area depending on how skewed the pressure profile is.
- Different contact solutions including normal pressures and shear tractions can be obtained depending on whether a constant load or displacement is specified in the normal and tangential directions. The evolution of the stick ratio is insensitive to the time-varying modulus and the rheological property of viscoelastic materials as long as the contact input (load or displacement) remains constant.
- However, the separation patterns of the stick and slip regions within the contacting area can be different even though the contact inputs are constant. This is determined by the contact phenomena encountered by the viscoelastic surface. When the same contact phenomenon (creep or stress relaxation) is experienced in both the normal and tangential directions, the evolution of the stick region follows a certain pattern. Otherwise, the way the stick and slip regions are separated would vary with time.
- Unlike that in an elastic solution, the transition from partial slip to gross sliding of viscoelastic materials tends to be abrupt under fully coupled conditions. Under the same contact inputs, a quicker transition can be achieved for a more fluid-like material. Compared to the case where a stress relaxation phenomenon is encountered in the normal direction, it requires a longer time for the viscoelastic surface to be in the gross sliding state when a creep phenomenon is encountered. Our findings suggest for the first time that a fully coupled contact condition leads to significantly different results than a semi-coupled one and that future analysis of the stick–slip or sliding of viscoelastic materials needs to be developed with consideration of fully coupled normal and tangential loads.

Although valid partial slip solutions can be achieved from the developed viscoelastic half-space model, films with finite thickness rather than a half-space, are the most common applications of viscoelastic materials, as stated by Zhang et al. [43]. A frictional contact model taking into account viscoelastic materials in the form of coatings or layers can provide helpful insights when designing a layer–substrate system. In addition, the modelling of frictional sliding or the rolling contact of viscoelastic surfaces remains a field that has not been exploited. These are the subjects of our current ongoing work. 

## Figures and Tables

**Table 1:** Parameters used in the simulation of a rigid sphere indenting a viscoelastic half-space (Maxwell model) under constant normal load.

Parameter	Value	Description (Unit)
R	3.5	Radius of the sphere (mm)
G η τ	81.92 81.92 1	Shear modulus of the spring in Maxwell model (GPa) Viscosity of the dashpot in Maxwell model (GPa·s) Relaxation time of the viscoelastic material (s)
ν	0.5	Poisson’s ratio of the viscoelastic material
$a_{0}$ $p_{0}$	117 3487	Hertzian contacting radius (μm) Hertzian peak normal pressure (MPa)

**Table 2:** Parameters used in the contact simulation of a rigid sphere indenting a viscoelastic half-space (Zener model) under constant normal load.

Parameter	Value	Description (Unit)
W	0.15	Input indentation load (N)
R	10	Radius of the sphere (mm)
$\mu_{\infty }$	3.86	Initial shear modulus of material (MPa)
$\frac{\mu_{\infty }}{\mu_{0}}$	2,4,8,10	Ratio of retardation time to relaxation time (ratio of initial shear modulus to modulus after infinite time)
τ	0.01	Relaxation time of the viscoelastic material (s)
ν	0.3	Poisson’s ratio of the viscoelastic material
$a_{0}$	588.7	Hertzian contacting radius for nondimensionalization (μm)
$p_{0}$	0.2067	Hertzian peak normal pressure for nondimensionalization (MPa)

**Figure A1:**
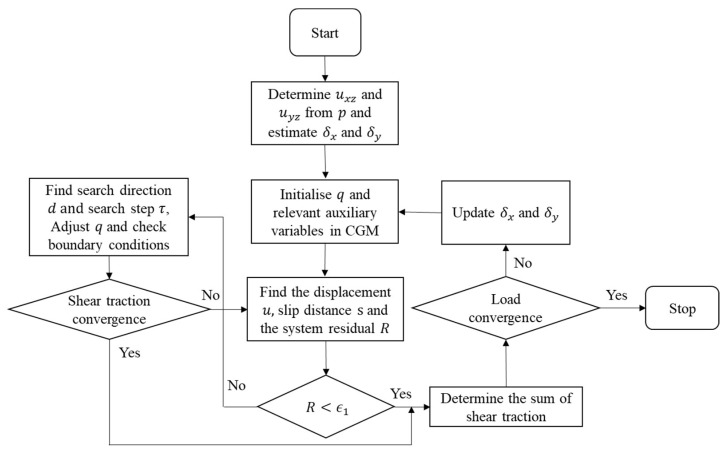
Flow chart of the algorithm for the tangential contact solver.

## Appendix A

The flow chart of the tangential contact solver for the elastic stick–slip problem is shown in Figure A1.
